# Associations between social isolation, withdrawal, and depressive symptoms in young adults: a cross-sectional study

**DOI:** 10.1186/s12888-025-06792-6

**Published:** 2025-04-03

**Authors:** Sujin Kim, Yun Seo Jang, Eun-Cheol Park

**Affiliations:** 1 Department of Public Health, Graduate School, Yonsei University, Seoul, Republic of Korea; 2 Institute of Health Services Research, Yonsei University, Seoul, Republic of Korea; 3 Health Insurance Review & Assessment Service, Wonju, Republic of Korea; 4 Department of Preventive Medicine, Yonsei University College of Medicine, Seoul, Republic of Korea

**Keywords:** Social isolation, Withdrawal, Isolation duration, Negative experiences, Depression

## Abstract

**Background:**

Social isolation and withdrawal, particularly among young people, have become significant social issues, raising concerns about mental health disorders. This study explores the association between social isolation, withdrawal, and depressive symptoms in young adults, focusing on sex differences and underlying factors.

**Methods:**

Data from 5,513 participants in the Seoul Government Survey on Socially Isolated and Withdrawn Young Adults were included in this study. Social isolation and withdrawal were measured based on levels of emotional or physical isolation and the amount of time spent at home instead of attending work or school. Depressive symptoms were assessed using the Patient Health Questionnaire (PHQ-9) scale. Multiple and multinomial logistic regression analyses were performed to investigate the associations between social isolation, withdrawal, and depression.

**Results:**

Socially isolated young adults demonstrated a strong association with depression (isolation only: Male, odds ratio [OR] 2.06, 95% confidence interval [CI] 1.38–3.08; Female, OR 2.95, 95% CI 2.06–4.95; isolation including withdrawal: Male, OR 2.56, 95% CI 1.11–5.89; Female, OR 2.40, 95% CI 1.04–5.57). However, withdrawal alone did not show any significant association. As depressive symptoms intensified (PHQ-9 ≥ 20), the association with social isolation strengthened (Male, OR 6.50, 95% CI 3.23–13.08; Female, OR 6.82, 95% CI 3.43–13.58). Prolonged isolation (≥ 3 years) was strongly associated with depression (Male, OR 2.91, 95% CI 1.76–4.79; Female, OR 6.04, 95% CI 3.58–10.20).

**Conclusion:**

Among young adults, the association between social isolation and depression intensifies with prolonged isolation and increased symptom severity, while withdrawal alone has no such effect. This highlights the importance of addressing social isolation and related issues in mental health interventions for young adults.

## Clinical trial number

Not applicable.

## Background

Social isolation and withdrawal behaviors have become significant social and economic issues, particularly among young people. Hikikomori, a phenomenon involving extreme social withdrawal, first emerged in Japan in the 1970s and gained prominence in the 1990s [1]. Following this, similar cases have been reported worldwide [2]. In South Korea, individuals who socially withdraw from society are often referred to as “reclusive loners.” This phenomenon first became an issue in the early 2000s amid intense academic competition. During this time, many adolescents began exhibiting signs of school refusal, withdrawal, and isolation, drawing attention to a broader phenomenon of socially withdrawn young adults. The competitive academic environment, particularly the pressures surrounding entrance exams, contributed significantly to these behaviors. Despite rapid industrialization and urbanization, South Korea remains a highly competitive society with strong educational pressures. Young people face significant academic and occupational stress as they strive to integrate themselves into society. Endless competition, high living costs, housing expenses, and potential risks such as COVID-19 have further impacted young people’s mental health and exacerbated feelings of loneliness and isolation [3, 4]. The concepts of social isolation and withdrawal are often used interchangeably as they are not entirely distinct. Social isolation refers to a lack of or limited social and emotional connections, whereas withdrawal involves a voluntary avoidance of social interactions. Although withdrawal can lead to isolation, not all socially isolated individuals exhibit withdrawal behaviors. Conversely, individuals may engage in withdrawal without necessarily being socially isolated. Considering these conceptual distinctions, this study classified participants into three groups—social isolation, social withdrawal, and a combined isolation-withdrawal—and analyzed their associations with depressive symptoms.

Recently, young individuals who have faced failure or frustration in highly competitive environments have increasingly turned to social withdrawal, with some even engaging in random acts of violence. These individuals are often diagnosed with mental illnesses, which reinforce the perception that social isolation is inherently linked to mental disorders. Conversely, social isolation and reclusion are increasingly recognized as distinct mental health issues. Some scholars view these behaviors as a modern form of depression, wherein individuals express feelings of depression and resentment while avoiding societal responsibilities and obligations [5]. Although the relationship between social withdrawal and mental health issues, such as depression, remains unclear, it is evident that social reclusion often accompanies mental health concerns [6]. Furthermore, conditions such as depression, anxiety, and mood disorders are closely linked to these behaviors [6–9]. Notably, depression is more prevalent among women [10], as they tend to be more vulnerable to mental health issues than men [11]. Therefore, considering sex differences is essential when examining the mental health implications of social isolation and withdrawal.

In South Korea, research on social isolation and reclusion has predominantly focused on the experiences of reclusive individuals [12] and the conceptual framework surrounding withdrawal behaviors [12–14]. However, few studies have investigated the relationship between social isolation and mental health. While some research has explored the connection between isolation and depression among older adults [15], a significant gap remains in understanding these issues among young adults. Despite growing societal interest in social withdrawal phenomena—such as hikikomori and reclusive behaviors—a clear consensus on the definitions of these terms has yet to emerge. Moreover, it remains uncertain whether isolation and withdrawal are conceptually distinct and whether they exhibit different associations with mental health. Furthermore, research on the underlying causes of these behaviors remains insufficient, highlighting the need for more comprehensive studies to explore the mental health implications of social isolation among young adults in South Korea.

Understanding the impact of social isolation on depression among young adults, particularly in Seoul—home to 21.9% of South Korea’s young adult population and a highly competitive job market—is crucial. This study investigates the relationship between social isolation and depressive symptoms among young adults in Seoul, South Korea, focusing on sex differences and various contributing factors.

## Methods

### Data sources and samples

This cross-sectional study utilized data from the Seoul Metropolitan Government’s Survey on Socially Isolated and Withdrawn Young Adults. The study sample was drawn from the young adult population distribution in Seoul using the 2022 Age-Specific Resident Registration Population Statistics provided by the Ministry of the Interior and Safety. Participants aged 19–39 years residing in Seoul completed self-reported online surveys between August 22, 2022, and September 23, 2022. Given the rarity of socially isolated and withdrawn individuals, additional survey links were distributed, with the cooperation of relevant institutions, to young people who had participated in support programs for social isolation and reclusion. The survey assessed participants’ social isolation and withdrawal tendencies (including employment status, duration and experience of reclusion and isolation, and past experiences of social withdrawal), lifestyle patterns, areas where support was needed, and general demographic information.

A sample of 5,000 individuals was established considering the sex and age distribution of the population based on the data from the Ministry of the Interior and Safety. Considering the rarity of socially isolated and withdrawn individuals, missing data for key variables were included, resulting in a total sample of 5,513 individuals (2,680 males and 2,833 females).

### Variables

The dependent variable was depressive symptoms, measured using the Korean version of the Patient Health Questionnaire-9 (PHQ-9). The PHQ-9 is a depression screening tool that comprises nine items, where respondents self-report their symptoms over the past two weeks, with scores ranging from 0 to 27. The Korean version of the PHQ-9, with a cutoff score of 10, demonstrated a sensitivity of 81.8% and specificity of 89.9%, indicating its reliability for detecting depressive symptoms [16]. In this study, Cronbach’s alpha coefficient for the PHQ-9 was 0.93, suggesting high reliability. The PHQ-9 was used in two ways: First, respondents were classified as having depressive symptoms based on a cutoff score of 10 [16, 17]. Second, depressive symptoms were further categorized such that respondents with a total score of < 5 were considered the normal group, and those scoring ≥ 5 were classified as the depressive symptom group. Specifically, individuals with a score of ≥ 5 but < 10 were categorized as having mild depressive symptoms, 10 to < 20 as moderate depressive symptoms, and ≥ 20 as severe depressive symptoms [18].

The variables of interest in this study were social isolation and withdrawal. These concepts have yet to reach societal consensus regarding their definitions, and the two terms—isolation and withdrawal—cannot be entirely distinguished as mutually exclusive. The scope of the study included young adults who were isolated, withdrawn, both isolated and withdrawn, or classified as high-risk.

Withdrawn young adults were defined as those who met al.l three of the following criteria: first, they rarely leave their home or room and mainly live indoors (e.g., “mostly stay home but go out occasionally for personal hobbies,” or “usually stay home but go out to nearby places such as convenience stores,” or “leave their room but not their home,” or “rarely leave their room”). Second, their withdrawal behavior persisted for at least six months. Third, they had not engaged in any economic activity in the past week nor pursued employment or education in the past month [19, 20].

Isolation was measured in terms of an individual’s social support network, focusing on the level of maintained emotional and physical connections. In Korean society, isolation is operationally defined by two criteria. First, the individual experiences either emotional or physical isolation. Emotional isolation is characterized by having no one to seek advice from during significant life challenges, request urgent help from, borrow money from in times of need, and confide in when feeling discouraged or depressed. Physical isolation refers to having little to no face-to-face interactions (once or twice a year at most) with people outside of family members, such as close friends, coworkers, or neighbors, excluding work-related interactions. Second, this state of isolation must have persisted for at least six months [19, 20].

Additionally, the study included some individuals who did not meet all the criteria for social isolation or reclusion but were considered high-risk, that is, those engaging in short-term economic activities out of necessity, experiencing isolation lasting three to six months, relying emotionally on family members while maintaining minimal connections outside the home, or holding jobs but cutting off all other social relationships.

Furthermore, this study included participants’ socioeconomic factors (such as sex, age, household income, education level, housing type, and homeownership), lifestyle behaviors (e.g., internet activity and frequency of leaving the house), behaviors related to isolation and withdrawal (including past experiences of isolation or exclusion and negative experiences before and after adulthood), and health status (such as self-reported health and the use of mental health medication) as variables.

### Statistical analysis

All analyses were stratified by sex based on the physiological differences between males and females [21]. The chi-square test was used to evaluate and compare the general characteristics of the study population. To examine the associations between social isolation, withdrawal, and depressive symptoms, we performed a multiple logistic regression analysis. In these analyses, we adjusted for potential confounders, including socioeconomic factors, lifestyle behaviors, behaviors related to isolation and withdrawal, and health status. Subgroup analyses were conducted based on the duration of social isolation and withdrawal, negative experiences in adulthood, and depressive symptom levels. All analyses included socioeconomic factors, lifestyle behaviors, and health status as covariates. The results are presented as odds ratios (ORs) and 95% confidence intervals (CIs). Multicollinearity was assessed using the variance inflation factor (VIF), and no multicollinearity issues were detected among the variables. The significance level was set at *p* < 0.05. Statistical analyses were performed using SAS version 9.4 (SAS Institute Inc., Cary, North, USA).

## Results

Table 1 presents the general characteristics of the study population. Among the 5,513 participants, 2,680 (48.6%) were males and 2,833 (51.4%) were females. In the male group, 148 participants experienced isolation only, 41 withdrawal only, and 38 experienced both isolation and withdrawal, with depressive symptoms observed in 54.1%, 48.8%, and 65.8% of participants, respectively. In the female group, 175 participants experienced isolation only, 50 withdrawal only, and 46 experienced both isolation and withdrawal, with depressive symptoms observed in 66.1%, 50.0%, and 50.0% of participants, respectively. Chi-squared analysis indicated a significant association between social isolation, withdrawal, and depressive symptoms. Furthermore, significant differences were observed in educational level, socioeconomic status, subjective health status, housing type, outdoor activity, past experiences of isolation or withdrawal, and negative experiences before and after adulthood in both male and female participants.

**Table 1:** General characteristics of study subjects

		Depressive symptoms											
	Male (*n* = 2680)							Female(*n* = 2833)					
	Total	Yes		No		*P* Value		Total	Yes		No		*P* Value
	**N**	**N**	**%**	**N**	**%**			**N**	**N**	**%**	**N**	**%**	
**Total(n = 5513)**	**2**,**680**	**775**	**(28.7)**	**1**,**925**	**(71.3)**			**2**,**833**	**758**	**(26.8)**	**2**,**075**	**(73.2)**	
**Social Isolation and withdrawal**						< 0.0001							< 0.0001
Isolation (only)	148	80	(54.1)	68	(45.9)			175	107	(61.1)	68	(38.9)	
Withdrawal (only)	41	20	(48.8)	21	(51.2)			50	25	(50.0)	25	(50.0)	
Isolation and withdrawal	38	25	(65.8)	13	(34.2)			46	23	(50.0)	23	(50.0)	
No	2,453	630	(25.7)	1,823	(74.3)			2,574	603	(23.4)	1,971	(76.6)	
**Age**						0.0353							0.4877
19 ~ 24	323	75	(23.2)	248	(76.8)			353	106	(30.0)	247	(70.0)	
25 ~ 29	668	211	(31.6)	457	(68.4)			1,042	279	(26.8)	763	(73.2)	
30 ~ 34	790	227	(28.7)	563	(71.3)			740	191	(25.8)	549	(74.2)	
35 ~ 39	899	242	(26.9)	657	(73.1)			698	182	(26.1)	516	(73.9)	
**Education**						< 0.0001							< 0.0001
Middle school or below	28	19	(67.9)	9	(32.1)			17	13	(76.5)	4	(23.5)	
High school	592	189	(31.9)	403	(68.1)			522	198	(37.9)	324	(62.1)	
College and above	2,060	547	(26.6)	1,513	(73.4)			2,294	547	(23.8)	1,747	(76.2)	
Socioeconomic level*						< 0.0001							< 0.0001
Low	828	268	(32.4)	560	(67.6)			882	291	(33.0)	591	(67.0)	
Middle	1,293	311	(24.1)	982	(75.9)			1,390	308	(22.2)	1,082	(77.8)	
High	522	158	(30.3)	364	(69.7)			518	136	(26.3)	382	(73.7)	
No answer	37	18	(48.6)	19	(51.4)			43	23	(53.5)	20	(46.5)	
**Health Condition**						< 0.0001							< 0.0001
Bad	351	186	(53.0)	165	(47.0)			428	228	(53.3)	200	(46.7)	
Average	1,119	293	(26.2)	826	(73.8)			1,214	281	(23.1)	933	(76.9)	
Good	1,210	276	(22.8)	934	(77.2)			1,191	249	(20.9)	942	(79.1)	
**Residential status**						< 0.0001							< 0.0001
Homeownership	1,305	316	(24.2)	989	(75.8)			1,292	294	(22.8)	998	(77.2)	
Rental	1,375	439	(31.9)	936	(68.1)			1,541	464	(30.1)	1,077	(69.9)	
**live alone**						0.435							0.2499
Yes	549	162	(29.5)	387	(70.5)			616	176	(28.6)	440	(71.4)	
No	2,131	593	(27.8)	1,538	(72.2)			2,217	582	(26.3)	1,635	(73.7)	
**Outdoor activity**						< 0.0001							< 0.0001
Often	2,310	617	(26.7)	1,693	(73.3)			2,409	584	(24.2)	1,825	(75.8)	
Sometimes	290	98	(33.8)	192	(66.2)			348	136	(39.1)	212	(60.9)	
Never	80	40	(50.0)	40	(50.0)			76	38	(50.0)	38	(50.0)	
**Internet activity**						0.034							0.9693
Yes	1,549	412	(26.6)	1,137	(73.4)			1,549	414	(26.7)	1,135	(73.3)	
No	1,131	343	(30.3)	788	(69.7)			1,284	344	(26.8)	940	(73.2)	
**Previous Experience of Isolation or withdrawal**						< 0.0001							< 0.0001
Yes	1,764	616	(34.9)	1,148	(65.1)			1,985	634	(31.9)	1,351	(68.1)	
No	916	139	(15.2)	777	(84.8)			848	124	(14.6)	724	(85.4)	
**Negative experience before adulthood**						< 0.0001							< 0.0001
Yes	1,378	692	(50.2)	686	(49.8)			2,157	710	(32.9)	1,447	(67.1)	
No	1,302	63	(4.8)	1,239	(95.2)			676	48	(7.1)	628	(92.9)	
**Negative Experiences in adulthood**						< 0.0001							< 0.0001
Yes	2,004	694	(34.6)	1,310	(65.4)			2,250	711	(31.6)	1,539	(68.4)	
No	676	61	(9.0)	615	(91.0)			583	47	(8.1)	536	(91.9)	
**Use of mental health medication**						< 0.0001							< 0.0001
Yes	231	170	(73.6)	61	(26.4)			242	152	(62.8)	90	(37.2)	
No	2,449	585	(23.9)	1,864	(76.1)			2,591	606	(23.4)	1,985	(76.6)	
Duration of isolation*						0.0653							< 0.0001
< 1 year	99	36	(36.4)	63	(63.6)			89	27	(30.3)	62	(69.7)	
1 year ~ < 3 years	47	17	(36.2)	30	(63.8)			57	25	(43.9)	32	(56.1)	
≥ 3 year	133	71	(53.4)	62	(46.6)			125	89	(71.2)	36	(28.8)	
No answer	2,401							2,562					
Duration of withdrawal*						0.4494							0.0381
< 1 year	142	46	(32.4)	96	(67.6)			193	69	(35.8)	124	(64.2)	
1 year ~ < 3 years	98	36	(36.7)	62	(63.3)			117	45	(38.5)	72	(61.5)	
≥ 3 year	130	56	(43.1)	74	(56.9)			114	60	(52.6)	54	(47.4)	
No answer	2,310							2,409					

* Included no answer

Table 2 illustrates the results of the multiple logistic regression analyses stratified by sex after adjusting for age, academic level, socioeconomic status, health condition, residential status, living arrangements, outdoor activity, Internet activity, previous experience of isolation or reclusion, negative experiences before adulthood, negative experiences in adulthood, and the use of psychiatric medication. Social isolation alone increases the odds of depressive symptoms by more than 2 times (male, OR 2.06, 95% CI 1.38–3.08; female, OR 2.95, 95% CI 2.06–4.25). Similarly, the combined state of isolation and withdrawal is associated with a twofold increase in the odds of depressive symptoms (male, OR 2.56, 95% CI 1.11–5.89; female. OR 2.40, 95% CI 1.04–5.57). However, withdrawal alone did not yield significant results.

**Table 2:** Results of factors associated with social isolation or reclusion and depressive symptoms

Variables	Depressive Symptoms			
	Male		Female	
	OR	95% CI	OR	95% CI
**Social isolation or withdrawal**				
Isolation (only)	2.06	(1.38-3.08)	2.95	(2.06-4.25)
Withdrawal (only)	1.04	(0.46-2.37)	0.92	(0.46-1.83)
Isolation and withdrawal	2.56	(1.11-5.89)	2.40	(1.04-5.57)
No	1.00		1.00	
**Age**				
19 ~ 24	1.00		1.00	
25 ~ 29	1.54	(1.05-2.26)	0.82	(0.59-1.13)
30 ~ 34	1.37	(0.92-2.03)	0.82	(0.58-1.15)
35 ~ 39	1.34	(0.90-2.00)	0.89	(0.63-1.25)
**Education**				
Middle school or below	4.51	(1.65-12.37)	5.32	(1.61-17.55)
High school	1.25	(0.96-1.64)	1.41	(1.09-1.81)
College and above	1.00		1.00	
**Socioeconomic level**				
Low	0.63	(0.46-0.85)	0.78	(0.58-1.06)
Middle	0.73	(0.56-0.96)	0.83	(0.63-1.08)
High	1.00		1.00	
No answer	1.05	(0.46-2.41)	1.49	(0.71-3.12)
**Health Condition**				
Bad	2.52	(1.85-3.43)	2.14	(1.62-2.82)
Average	1.11	(0.89-1.39)	0.92	(0.74-1.14)
Good	1.00		1.00	
**Residential status**				
Homeownership	1.00		1.00	
Rental	1.39	(1.13-1.71)	1.07	(0.87-1.32)
**live alone**				
Yes	1.01	(0.79-1.30)	1.07	(0.84-1.36)
No	1.00		1.00	
**Outdoor activity**				
Often	1.00		1.00	
Sometimes	0.97	(0.69-1.35)	1.40	(1.05-1.86)
Never	2.21	(1.25-3.91)	1.62	(0.91-2.88)
**Internet activity**				
Yes	1.00		1.00	
No	1.49	(1.22-1.83)	1.23	(1.01-1.48)
**Previous Experience of isolation or withdrawal**				
Yes	1.82	(1.45-2.30)	1.59	(1.26-2.00)
No	1.00		1.00	
**Negative experiences before adulthood**				
Yes	2.98	(2.17-4.08)	3.23	(2.30-4.53)
No	1.00		1.00	
**Negative experiences in adulthood**				
Yes	2.61	(1.87-3.63)	2.61	(1.83-3.71)
No	1.00		1.00	
**Use of psychiatric medication**				
Yes	1.00		1.00	
No	0.15	(0.11-0.21)	0.28	(0.21-0.38)

Table 3 presents the results of the subgroup analysis based on isolation and withdrawal statuses. Isolation lasting less than 1 year increased the odds of depressive symptoms (OR 2.13, 95% CI 1.00–4.55), while prolonged isolation of ≥ 3 years showed a strong association (OR 6.04, 95% CI 3.58–10.20) in female participants. Male participants displayed a similar pattern, with prolonged isolation increasing the odds of depressive symptoms by 2.91 times (95% CI 1.76–4.79). Female participants who experienced isolation during adolescence had significantly higher odds of developing depressive symptoms (OR 8.41, 95% CI 2.40–29.43). Experiencing isolation for the first time in the late 20s, in contrast to the early 20s, was associated with higher odds of depressive symptoms in both sexes, with a particularly strong effect observed in female participants (male, OR 2.33, 95% CI 1.19–4.57; female, OR 2.90, 95% CI 1.57–5.36). Conversely, neither the duration of withdrawal nor the age at first withdrawal was significantly associated with depressive symptoms. Family conflict, domestic violence, or parental divorce as reasons for isolation or withdrawal were associated with the highest odds of depressive symptoms (male, OR 5.47, 95% CI 2.55–11.74; female, OR 3.23, 95% CI 1.81–5.76), as were psychological or physical health issues (male, OR 3.29, 95% CI 1.86–5.82; female, OR 3.05, 95% CI 1.94–4.79).

**Table 3:** Subgroup analysis stratified by social isolation and withdrawal

Variables	Depressive Symptoms			
	Male		Female	
	OR	95% CI	OR	95% CI
**Duration of isolation**				
< 1 year	2.14	(0.94-4.86)	2.13	(1.00-4.55)
1 year ~ < 3 years	1.4	(0.65-3.01)	0.71	(0.88-3.30)
≥ 3 year	2.91	(1.76-4.79)	6.04	(3.58-10.20)
**Duration of withdrawal**				
< 1 year	1.34	(0.31-5.78)	1.49	(0.50-4.46)
1 year ~ < 3 years	1.23	(0.44-3.44)	0.5	(0.19-1.31)
≥ 3 year	1.73	(0.77-3.92)	1.45	(0.65-3.23)
**First isolation age**				
< 19	3.14	(0.84-11.81)	8.41	(2.40-29.43)
19 ~ 24	1.97	(1.02-3.79)	2.71	(1.46-5.03)
25 ~	2.33	(1.19-4.57)	2.9	(1.57-5.36)
**First withdrawal age**				
< 19	0.75	(0.07-7.74)	0.62	(0.16-2.50)
19 ~ 24	1.27	(0.50-3.19)	0.82	(0.34-2.01)
25 ~	1.4	(0.54-3.63)	1.31	(0.58-2.97)
**Reasons for social isolation or withdrawal†**				
Economic and Academic Difficulties	1.87	(1.20-2.93)	2.09	(1.37-3.19)
Social Relationship and Interpersonal Conflicts	2.53	(1.58-4.05)	2.44	(1.62-3.69)
Family Conflicts and Domestic Issues	5.47	(2.55-11.74)	3.23	(1.81-5.76)
Psychological and Physical Health Problems	3.29	(1.86-5.82)	3.05	(1.94-4.79)

† multiple answers

Table 4 presents the subgroup analysis results on the associations between depressive symptoms and negative experiences before and during adulthood. Experiences before adulthood, such as caregiver violence or verbal humiliation (OR 2.01, 95% CI 1.66–2.44) and frequent moves or school changes (OR 2.00, 95% CI 1.63–2.45), were linked to higher depressive symptoms risk in female participants. For male participants, bullying or peer exclusion at school or in the neighborhood was significantly associated with depressive symptoms (OR 2.79, 95% CI 2.27–3.42). In adulthood, male and female participants who left school owing to financial difficulties (male, OR 4.08, 95% CI 3.06–5.44; female, OR 3.10, 95% CI 2.32–4.15) or faced career-related pressure from close individuals (male, OR 2.94, 95% CI 2.37–3.64; female, OR 2.87, 95% CI 2.34–3.51) had over twice the odds of depressive symptoms.

**Table 4:** Subgroup analysis stratified by negative experiences

Variables	Depressive Symptoms			
	Male		Female	
	OR	95% CI	OR	95% CI
**Negative experience before adulthood†**				
No	1.00		1.00	
Sudden economic hardship within the household	1.03	0.840.-1.26)	1.46	(1.20-1.77)
Severe physical punishment or verbal abuse by a parent or caregiver	2.11	(1.72-2.58)	2.01	(1.66-2.44)
Emotional distress (such as anxiety or depression) in a family member	1.94	(1.59-2.37)	1.82	(1.50-2.22)
The loss of a close person	1.49	(1.22-1.45)	1.87	(1.55-2.27)
Frequent relocations or school transfers	1.73	(1.40-2.13)	2.00	(1.63-2.45)
Instances of bullying or social exclusion within school or the community	2.79	(2.27-3.42)	1.66	(1.37-2.01)
**Negative experience in adulthood†**				
No	1.00		1.00	
Discontinue education due to financial constraints	4.08	(3.06-5.44)	3.10	(2.32-4.15)
Career coercion or employment pressure from a close person	2.94	(2.37-3.64)	2.87	(2.34-3.51)
Failure in university entrance	1.68	(1.21-2.33)	1.45	(1.04-2.02)
Failure to secure desired employment	1.26	(1.02-1.57)	1.22	(0.98-1.52)
Deception or betrayal by a trusted individual	1.73	(1.41-2.12)	2.02	(1.66-2.47)
Loss of a close relationship (separation, death)	1.36	(1.11-1.67)	1.58	(1.29-1.94)

† multiple answers

**Fig. 1:**
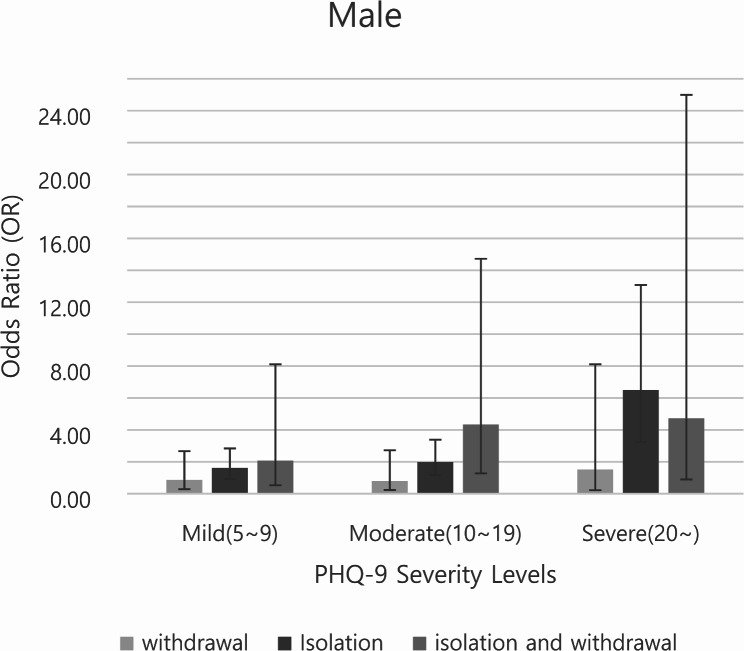
Male subgroup analysis stratified by PHQ-9 scores (reference: PHQ-9 < 5) † The vertical axis represents the odds ratio (OR), and the error bars indicate the 95% confidence intervals (95% CI)

**Fig. 2:**
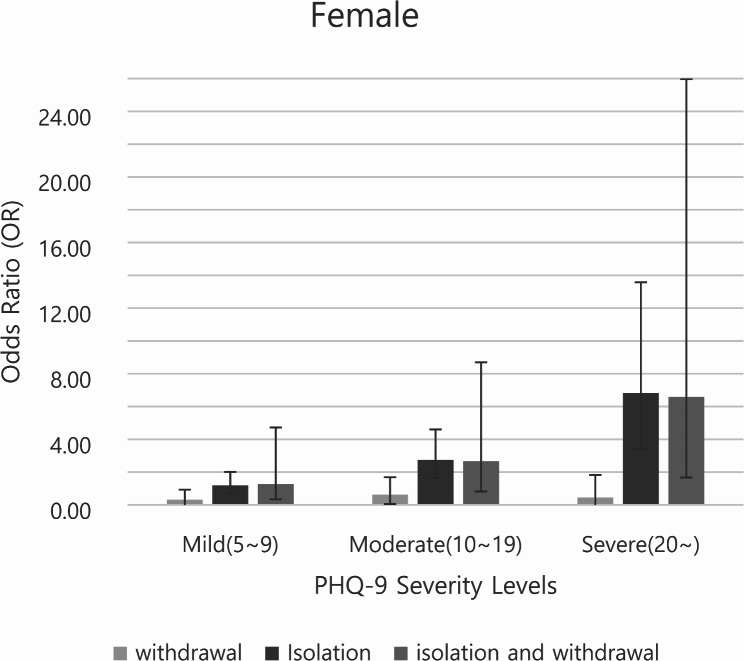
Female subgroup analysis stratified by PHQ-9 scores (reference: PHQ-9 < 5) † The vertical axis represents the odds ratio (OR), and the error bars indicate the 95% confidence intervals (95% CI)

Figures 1 and 2 illustrate the subgroup analysis of the dependent variables for male and female participants, respectively, with the error bars representing the 95% confidence intervals. Analyses of the PHQ-9 scores revealed that the ORs tended to increase with the severity of depressive symptoms. Social isolation was associated with significantly higher odds of experiencing severe depression in both male and female participants (male, OR 6.50, 95% CI 3.23–13.08; female, OR 6.82, 95% CI 3.43–13.58).

## Discussion

This study examined the relationships between social isolation, withdrawal, and depressive symptoms in young adults. Socially isolated young adults had a higher association with depression (PHQ-9 ≥ 10) compared to their non-isolated peers. Notably, as depressive symptom severity increased, the strength of this association also tended to rise, with the link between social isolation and severe depression (PHQ-9 ≥ 20) appearing strong. These trends were similar for cases of isolation that included withdrawal, whereas withdrawal alone showed no significant association. Prolonged isolation was more strongly linked to depressive symptoms than early isolation, with a particularly strong effect in female participants. Initial experiences of isolation during adolescence were associated with substantially higher odds of depressive symptoms, which was particularly pronounced among female participants. This relationship remained statistically significant even after controlling for demographic factors, lifestyle behaviors, and mental health variables.

Several studies have explored the relationships between social isolation, withdrawal, and mental health [22–24]. In particular, individuals who experience social withdrawal are at a higher risk of depression, not only among older adults and adolescents but also among young adults [25, 26]. Furthermore, early experiences of isolation during childhood or adolescence are associated with depression or suicidal tendencies in adulthood [27, 28]. In South Korea, an association between social withdrawal and depression has been observed in both older and young adults [9, 29].

Given the lack of clear societal consensus on the definitions of isolation and withdrawal, with withdrawal often considered a subset of isolation, this study categorized participants into isolation, withdrawal, and combined isolation-withdrawal groups. The analysis demonstrated the substantial impact of isolation on mental health. Individuals in a state of isolation without withdrawal, as well as those experiencing both isolation and withdrawal, showed a strong association with depressive symptoms across sexes, with this effect being particularly pronounced in cases of prolonged isolation. When individuals lack reliable social support or in-person interactions, the impact of isolation significantly contributes to depressive symptoms [6]. Conversely, simply staying at home and avoiding outings may not necessarily be directly associated with mental health issues. In cases where individuals experience social or familial pressures or when feelings of social alienation or discomfort prompt them to withdraw from their surroundings, this lifestyle may offer a sense of relief [30]. Furthermore, unsociable individuals who naturally prefer solitude can experience life satisfaction, indicating that various factors, including personal dispositions and motivations behind seeking solitude, may be interwoven and ultimately related to their mental health outcomes [31].

According to the findings of this study, depressive symptoms were generally more prevalent among female participants, which is consistent with previous research showing a strong association between depressive symptoms and being female [10, 32]. However, among socially withdrawn and isolated young adults, males also exhibit a higher likelihood of experiencing severe depression. This may be attributed to a greater tendency for social isolation among males than females [33].

The duration of isolation (including combined isolation and withdrawal) tended to show a decrease in depressive symptoms between one and three years compared to less than one year; however, the likelihood of depression significantly increased with isolation extending beyond three years. While avoiding social relationships and participation does not necessarily equate to mental illness, prolonged isolation can exacerbate or contribute to the development of mental health problems [34]. Nevertheless, this trend was not statistically significant during the withdrawal period (including for the combined treatment). Female participants whose initial experience of isolation occurred during adolescence were more likely to experience depression.

A significant finding was that similar to employment or academic failure, family conflict or psychological and physical health issues are major contributors to isolation and withdrawal and are closely associated with depression. While many socially isolated individuals cited job or academic failure as reasons for their isolation, underlying issues, such as family conflict and health problems, may have stronger associations with depression [35–37]. Furthermore, social isolation can lead to employment and academic failure and exacerbate family conflicts, thereby increasing the likelihood of depression [38, 39]. This suggests that social isolation not only directly affects depressive symptoms but also indirectly influences them through economic and familial stressors. Furthermore, socioeconomic factors and lifestyle variables may act as covariates that influence the magnitude of the association between isolation, withdrawal, and depression, potentially moderating or mediating this relationship [40, 41]. For example, individuals with low socioeconomic status are more likely than those with high economic status to experience isolation, which in turn may increase their risk of depression [42]. Additionally, the strength and direction of this association may vary depending on age [43]. To elucidate these pathways more clearly, future research should employ mediation analysis to explore the multifaceted impact of social isolation on mental health.

In this study, negative pre-adulthood experiences were strongly linked to mental health in adulthood, suggesting that these early experiences may be causal factors for current depressive symptoms. For women, violence and abuse by a caregiver or frequent school changes and relocations are factors associated with depressive symptoms [44, 45]. This suggests that emotional and environmental instability are closely linked to psychological issues in women. For men, the primary factor influencing depressive symptoms is social exclusion through bullying or ostracism [46]. These findings are consistent with previous research showing that early trauma has long-term effects on mental health and social functioning [47]. In adult experiences, economic factors and job-related coercive issues were associated with depressive symptoms in both men and women [48].

Furthermore, while financial and career problems were more strongly linked to depressive symptoms among the young population, socially isolated and withdrawn young adults showed that underlying issues such as family conflicts and health problems were more directly related to their depressive symptoms than the financial issues cited. This finding suggests that the causes and patterns of depressive symptoms differ among the general young adult population and those who are socially isolated or withdrawn. Further research is required to explore these differences.

Interventions for socially isolated and withdrawn young adults should prioritize efforts to address social isolation. Additionally, interventions should focus on resolving underlying issues, such as psychological and health-related problems. While economic support and job opportunities can help prevent isolation and withdrawal, addressing the fundamental causes of these issues is essential for improving young adults’ mental health.

This study has several limitations. First, its cross-sectional design limited the ability to establish causal relationships between social isolation, withdrawal, and depressive symptoms. To address this, future studies should apply a longitudinal design to better determine these causal relationships. Second, the data were drawn from a sample of young adults residing in Seoul, which limits the generalizability of the findings. However, the high concentration of young adults living in Seoul and the likelihood of a significant proportion of socially isolated or withdrawn young adults residing in urban areas were considered. Future research should expand the study to include diverse regions beyond major cities to enhance the generalizability of the findings. Third, the classification criteria for social isolation and withdrawal may be ambiguous, and the lack of a clear consensus on these definitions is a notable limitation. Additionally, as these data were collected as part of administrative efforts to identify and support socially isolated individuals, the dataset includes those who have been socially isolated for ≥ 6 months and individuals in the high-risk category, such as those who have experienced isolation for 3 months or those with fluctuating isolation patterns. Fourth, although stratified sampling was used, the survey was administered primarily to a panel selected by the survey institution, which introduced a potential selection bias.

Despite these limitations, this study has several strengths. It used large-scale data from the first extensive survey conducted by the Seoul Metropolitan Government on socially isolated and withdrawn young adults. This dataset is particularly valuable because it addresses the challenges of surveying this population, who are often difficult to reach or interview because of the nature of their isolation.

## Conclusions

This study identified a significant association between social isolation, withdrawal, and depressive symptoms in young adults, with social isolation having a more pronounced effect on depressive symptoms than withdrawal. Prolonged isolation was strongly linked to the severity of depressive symptoms, particularly among female participants. Additionally, this association tended to increase with the severity of depressive symptoms. Furthermore, isolation alone consistently showed a significant association with depressive symptoms, whereas withdrawal alone did not demonstrate a significant effect. In interventions for socially isolated and withdrawn young adults, it is essential to address social isolation and underlying issues such as psychological and health-related problems to improve mental health.

## Data Availability

The data supporting the conclusions of this article are available from the Seoul Government Survey on Socially Isolated and Withdrawn Young Adults in 2022 repository, https://data.seoul.go.kr/dataList/OA-22347/F/1/datasetView.do; https://www.data.go.kr/data/15114298/fileData.do.

## Abbreviations

COVID-19: coronavirus disease
OR: odds ratio
CI: confidence interval
PHQ-9: Patient Health Questionnaire-9
